# Microbial regulation of global biogeochemical cycles

**DOI:** 10.3389/fmicb.2014.00103

**Published:** 2014-03-14

**Authors:** Johannes Rousk, Per Bengtson

**Affiliations:** Department of Biology/Microbial Ecology, Lund UniversityLund, Sweden

**Keywords:** microbial ecology, biogeochemistry, stoichiometry, climate change, soil microbiology, elemental fluxes, respiration, aquatic microbiology

Global biogeochemical cycles of carbon and other nutrients are increasingly affected by human activities (Griggs et al., 2013). So far, modeling has been central for our understanding of how this will affect ecosystem functioning and the biogeochemical cycling of elements (Treseder et al., 2012). These models adopt a reductive approach built on the flow of elements between pools that are difficult or even impossible to verify with empirical evidence. Furthermore, while some of these models include the response in physiology, ecology and biogeography of primary producers to environmental change, the microbial part of the ecosystem is generally poorly represented or lacking altogether (Stein and Nicol, 2011; Treseder et al., 2012).

The principal pool of carbon and other nutrients in soil is the organic matter (Schimel, 1995). The turnover time of this reservoir is governed by the rate at which microorganisms consume it. The rate of organic matter degradation in a soil is determined by both the indigenous microbial community and the environmental conditions (e.g., temperature, pH, soil water capacity, etc.), which govern the biogeochemical activities of the microorganisms (Waksman and Gerretsen, 1931; Schmidt et al., 2011). The dependences of these biogeochemical activity rates on environmental conditions such as pH, moisture and temperature have been frequently studied (Conant et al., 2011; Schmidt et al., 2011). However, while various microorganisms involved in carrying out biogeochemical processes have been identified, biogeochemical process rates are only rarely measured together with microbial growth, and one of the biggest challenges for advancing our understanding of biogeochemical processes is to systematically link biogeochemistry to the rate of specific metabolic processes (Rousk and Bååth, 2011; Stein and Nicol, 2011). We also need to identify the factors governing these activities and if it results in feedback mechanisms that alter the growth, activity and interaction between primary producers and microorganisms (Treseder et al., 2012). By determining how different groups of microorganisms respond to individual environmental conditions by allocating e.g. carbon to production of biomass, CO_2_ and other products, a mechanistic as well as quantitative understanding of formation and decomposition of organic matter, and the production and consumption of greenhouse gases, can be achieved.

In this Research Topic, supported by the Swedish research councils' program “Biodiversity and Ecosystem Services in a Changing Landscape” (BECC), we intend to promote an alternative framework to address how cycling of carbon and other nutrients will be altered in a changing environment from the first-principle mechanisms that drive them—namely the ecology, physiology and biogeography of microorganisms. In order to improve the predictive power of current models, the alternative framework supports the development of new models of biogeochemical cycles that factor in microbial physiology, ecology, and biogeochemistry. Our ambition has been richly rewarded by an extensive list of submissions. We are pleased to present contributions including primary research targeting the microbial control of biogeochemistry, comprehensive reviews of how microbial processes and communities relate to biogeochemical cycles, identification of critical challenges that remain, and new perspectives and ideas of how to optimize progress in our understanding of the microbial regulation of biogeochemistry.

Our Research Topic presents new findings about the importance of the microbial community composition, their metabolic state, and the activity of enzymes for the fate and degradation of specific substrates such as chitin (Beier and Bertilsson, 2013), the degradation of more complex compounds such as those constituting plant litter (Moorhead et al., 2013; Rinkes et al., 2013), and the metabolism and biogeochemical cycling of one-carbon compounds (Aronson et al., 2013; Basiliko et al., 2013; Kappler and Nouwens, 2013). The environmental control and land-use perturbation of microbial communities and methane production were assessed in a comprehensive review (Aronson et al., 2013) as well as a case study (Basiliko et al., 2013) and a meta-analysis (Holden and Treseder, 2013). Other contributions have focused on how environmental variables that are affected by climate change can modulate microbial activities by e.g. their influence on the production and activity of enzymes (Steinweg et al., 2013), while Bradford (2013) has provided a comprehensive review of how microbial processes respond to warmer temperatures. These reviews are accompanied by a new suggestion for how we can achieve better predictions for microbial responses (and feedbacks) to climate change (de Vries and Shade, 2013), while Moorhead et al. (2013) identify knowledge gaps and provide important insights about how data on microbial communities, environmental conditions, and enzyme activities can be used to better inform enzyme-based models.

Several submissions have highlighted the importance for plant-microbial feedbacks for the regulation of organic matter decomposition and formation (Moorhead et al., 2013; Thomson et al., 2013; Churchland and Grayston, under review), the production of biogenic volatile organic compounds (Rinnan et al., 2013), and the community composition of methanogens and sulfate reducing bacteria (Zeleke et al., 2013). A very active research area in soil microbial ecology is presently how small amounts of labile carbon sources can trigger, or “prime,” the decomposition of soil organic matter. A route toward a more general understanding of the regulation of plant-soil interaction for biogeochemistry, that may well facilitate our understanding of “priming effects,” could be the incorporation of stoichiometric concepts (Dijkstra et al., 2013; Mooshammer et al., 2014). Stoichiometric variations in the concentration of nutrients, combined with variations in carbon and nutrient demands of different decomposer groups, also seems to be reflected in the degradation rate of plant litter (Rinkes et al., 2013). A comprehensive review of biogenic fixation of nitrogen demonstrates the importance of interactions between different biogeochemical cycles for nitrogen fixation in ecosystems with nitrogen-limited plant productivity (Rousk et al., 2013). These contributions emphasize that stoichiometric variations in nutrient concentrations are of importance for both factors that could determine the propensity for organic matter to accumulate in an ecosystem, and thus for carbon to be sequestered.

Some contributions to this Research Topic have also highlighted methodological challenges that urgently need attention. For instance, the ability of contemporary isotopic tracer methods to estimate microbial contributions to biogeochemical processes could be systematically overestimated (Hobbie and Hobbie, 2013), suggesting that estimates of the turnover of low molecular weight organic compounds, and possibly also for estimations of nitrogen transformation rates, need to be revised. Additionally, there is a need to move from laboratory-based estimations of the microbial role in ecosystem level processes, often omitting crucial components such as the presence of plants, to field-based assessments in intact systems (Rinkes et al., 2013).

The contributions to our Research Topic have opened up new horizons and stimulated conceptual developments in our basic understanding of the regulating factors of global biogeochemical cycles. Within this forum, we have begun to bridge Microbial Ecology and Biogeochemistry, connecting microbial activities at the microcosm scale to carbon fluxes at the ecosystem-scale, and linking above- and belowground ecosystem functioning. We are hopeful that we have initiated conceptual developments that can reach far beyond this Research Topic. It is a mere first step, but we are confident it is directed toward a predictive understanding of the microbial regulation of global biogeochemical cycles.
